# *Aspergillus* section *Flavi* and Aflatoxins: Occurrence, Detection, and Identification in Raw Peanuts and Peanut-Based Products Along the Supply Chain

**DOI:** 10.3389/fmicb.2019.02602

**Published:** 2019-11-22

**Authors:** Mahror Norlia, Selamat Jinap, Mahmud Ab Rashid Nor-Khaizura, Son Radu, Nik Iskandar Putra Samsudin, Farah Asilah Azri

**Affiliations:** ^1^Faculty of Food Science and Technology, Universiti Putra Malaysia, Serdang, Malaysia; ^2^School of Industrial Technology, Universiti Sains Malaysia, Penang, Malaysia; ^3^Institute of Tropical Agriculture and Food Security, Universiti Putra Malaysia, Serdang, Malaysia

**Keywords:** aflatoxins, *Aspergillus* section *Flavi*, peanuts, peanut supply chain, raw peanuts, peanut-based products

## Abstract

Aflatoxin contamination in foods is a global concern as they are carcinogenic, teratogenic and mutagenic compounds. The aflatoxin-producing fungi, mainly from the *Aspergillus* section *Flavi*, are ubiquitous in nature and readily contaminate various food commodities, thereby affecting human’s health. The incidence of aflatoxigenic *Aspergillus* spp. and aflatoxins in various types of food, especially raw peanuts and peanut-based products along the supply chain has been a concern particularly in countries having tropical and sub-tropical climate, including Malaysia. These climatic conditions naturally support the growth of *Aspergillus* section *Flavi*, especially *A. flavus*, particularly when raw peanuts and peanut-based products are stored under inappropriate conditions. Peanut supply chain generally consists of several major stakeholders which include the producers, collectors, exporters, importers, manufacturers, retailers and finally, the consumers. A thorough examination of the processes along the supply chain reveals that *Aspergillus* section *Flavi* and aflatoxins could occur at any step along the chain, from farm to table. Thus, this review aims to give an overview on the prevalence of *Aspergillus* section *Flavi* and the occurrence of aflatoxins in raw peanuts and peanut-based products, the impact of aflatoxins on global trade, and aflatoxin management in peanuts with a special focus on peanut supply chain in Malaysia. Furthermore, aflatoxin detection and quantification methods as well as the identification of *Aspergillus* section *Flavi* are also reviewed herein. This review could help to shed light to the researchers, peanut stakeholders and consumers on the risk of aflatoxin contamination in peanuts along the supply chain.

## Introduction

Mycotoxins are toxic secondary metabolites produced mostly by fungi from the genus *Aspergillus*, *Penicillium*, *Fusarium*, and *Alternaria* which are formed pre- and post-harvest (Pitt and Hocking, 2009). The most significant mycotoxins contaminating agricultural commodities and foods are aflatoxins, fumonisins, ochratoxin A, zearalenone, patulin, citrinin, and deoxynivalenol (Afsah-Hejri et al., 2013a). According to Wild and Turner (2002), of these, aflatoxins are the most toxic, and have been extensively studied.

Peanuts (*Arachis hypogaea* L.) are legumes native to the western hemisphere. It is believed that peanut cultivation began in Bolivia and its neighboring countries before traders spread it to Asian and African continents. Peanuts consist of kernels and protective layer of outer shells. Peanuts are a good source of total energy, fats, minerals, vitamins, and proteins (Singh and Singh, 1991). Presently, peanuts are well adapted and widely grown in the tropical and sub-tropical countries such as India, China, Nigeria, Kenya, and the Southeast Asian countries including Malaysia (Archer, 2016). However, peanuts are not the main agricultural commodities in Malaysia, and the people rely on the import of peanuts from other countries such as India, China and Vietnam to fulfill the increasing demand (Afsah-Hejri et al., 2013a).

Recently, the occurrence of *Aspergillus* section *Flavi* and aflatoxin contamination has been reported in the supply chain of peanut-importing countries including Malaysia (Guezlane-tebibel et al., 2013; Norlia et al., 2018b). As a peanut-importing country, Malaysia is more concerned about aflatoxin production and contamination during storage, since Malaysia’s tropical weather favors the growth of fungi including that of the aflatoxigenic *Aspergillus* spp. In addition, the precise identification and characterization of aflatoxigenic *Aspergillus* spp. that could survive and proliferate on the imported peanuts are less studied as compared to that on peanuts in the field (Zhang et al., 2017).

## Aflatoxins and *Aspergillus* section *Flav*i

To date, there are 18 known analogs of aflatoxins with three series being significantly important from a food safety perspective: B-series (AFB_1_ and AFB_2_), G-series (AFG_1_ and AFG_2_) and M-series (AFM_1_ and AFM_2_). *A. flavus* and *A. parasiticus* are the major producers of aflatoxins, whereby the *A. flavus* produce B-series aflatoxins, while *A. parasiticus* produce both B- and G-series. The “B” and “G” refer to the blue and green fluorescence colors produced under UV light, while the subscript numbers indicate major and minor compounds, respectively (Dhanasekaran et al., 2011). Of these, AFB_1_ is classified as a Group 1 carcinogen by the IARC (1993) due to the sufficient evidence of its involvement in cancer development in humans. Upon ingestion of the contaminated feeds by the animals, AFB_1_ and AFB_2_ are then metabolized in the body, thereby causing milk produced by the animals to be contaminated with their hydroxylated derivatives known as AFM_1_ and AFM_2_ (Dhanasekaran et al., 2011).

Morphological identification of *Aspergillus* section *Flavi* is usually based on the microscopic structures, such as the uni- or biseriate conidial heads, production of dark-colored sclerotia by certain species, and yellow green to brown shades conidia. *Aspergillus* section *Flavi* includes 33 species, and most of them are natural producers of aflatoxins (Frisvad et al., 2019). Members of this section can exist in the soil as sclerotia or conidia, or mycelia in plant tissue. Sclerotia of *A. flavus* (Horn et al., 2009a) and *A. parasiticus* (Horn et al., 2009b) can also be produced naturally in crops by an asexual or sexual stage and are dispersed onto the soil during harvest. Sclerotia can survive under severe environmental conditions in the field and germinate into mycelia, followed by the formation of the conidiophores and conidia when the condition becomes favorable (Horn et al., 2014). The mechanism of *A. flavus* sexual reproduction in a natural environment which includes the fertilization in soil and crops, has been described by Horn et al. (2016). The exchange of genetic materials during sexual recombination results in the high genetic diversity in *A. flavus* population. Thus, the morphology, mycotoxin production and vegetative compatibility groups (VCGs) in *A. flavus* are more diverse as compared to other species in section *Flavi*.

According to Cotty (1989), two morphotypes of *A. flavus* have been designated based on the size of their sclerotia. The large (L) strain and small (S) strain are indicated by sclerotia size of >400 μm and <400 μm in diameter, respectively. The S-type *A. flavus* has been reported to be more toxigenic than the L-type, and it is also more dominant in the West Africa. Probst et al. (2007) revealed that the S-type *A. flavus* was the causal agent of the aflatoxicosis outbreak in Kenya in 2004 due to the consumption of contaminated corn. However, the phylogenetic studies revealed that the S-strain *A. flavus* from Kenya were different from the US and Asian S-type *A. flavus*, but were closer to *A. minisclerotigenes* (Probst et al., 2012).

The accurate identification of *Aspergillus* section *Flavi* requires a triphasic approach which includes the morphological, chemical and molecular approaches as these species are closely related and could not be easily distinguished by morphological characteristics alone (Varga et al., 2011; Frisvad et al., 2019). The information on the production of secondary metabolites such as cyclopiazonic acid (CPA), aspergillic acid, kojic acid, asperfuran, paspalinin, paspaline, nominine, chrysogine, parasiticolides, aflavarins, aflatrems, and aflavinines will strengthen the species identification (Pildain et al., 2008; Varga et al., 2011; Frisvad et al., 2019). According to Lansden and Davidson (1983), CPA can be found either alone or co-occurring with aflatoxins in various crops such as peanuts and corn. During the outbreak of Turkey X disease in England (1960’s), about 100,000 of Turkeys and other poultry died due to the consumption of contaminated peanut meal imported from Brazil. It was believed that CPA acted as a co-contaminant with aflatoxins, thereby causing severe aflatoxicosis (Cole, 1986). The co-occurrence of CPA and aflatoxins in stored peanuts has also been reported by Zorzete et al. (2013).

In contrast, *A. sojae* and *A. oryzae*, which are respectively known as the domesticated counterparts of *A. parasiticus* and *A. flavus*, do not produce aflatoxins, although they possess the homologues of the aflatoxin biosynthesis pathway gene. For their safety status, these species are widely used for food fermentation in Asian countries such as *sake*, soy sauce and *miso* (Payne et al., 2006). There are also some cases of *A. flavus* losing their toxigenic properties thus becoming non-aflatoxigenic even though they possess all the necessary genes for aflatoxin biosynthesis in their genome (Yu et al., 2004). It is believed that the genetic variation in the non-aflatoxigenic *A. flavus* strains is caused by the sexual reproduction and genetic recombinant in nature (Horn et al., 2016).

The non-aflatoxigenic *A. flavus* has been previously described and is used as a biological control agent based on the competitive exclusion to reduce the aflatoxigenic species in peanuts (Chulze et al., 2014; Ehrlich, 2014). The conidia of the inoculated non-aflatoxigenic strains will compete with the aflatoxigenic strains naturally present in the soil for growth and essential nutrients from peanuts. The application of non-aflatoxigenic *A. flavus* in the peanut field successfully reduced the aflatoxin contamination in peanut-producing regions in the United States (Dorner et al., 2003) and Northern Argentina (Zanon et al., 2016). In addition, Dorner and Cole (2002) also successfully demonstrated the ability of non-aflatoxigenic strains of *A. flavus* and *A. parasiticus* to reduce the aflatoxin contamination in peanuts during storage. However, there is a limitation on using the non-aflatoxigenic strains as a biocontrol. According to Ehrlich (2014), the application of non-aflatoxigenic strains in the field should be of concern as the outcross with the native population of *A. flavus* in soil could result in the offspring regaining the ability to produce aflatoxins. The global warming that causes the climate change might also be a challenge as the crops can be subjected to damage and further facilitate the fungal infection since the stress on plants could induce the gene expression for mycotoxin production and sexual recombination in *A. flavus*.

## Factors Affecting *Aspergillus* spp. Growth and Aflatoxin Production in Peanuts

Temperature, relative humidity and moisture content are the main factors that determine the ability of *A. flavus* to grow during storage (Waliyar et al., 2015a). Relative humidity and water activity (a_w_) in foods are interrelated to each other and could be used to determine the ability of fungi to grow. Technically, a_w_ is defined as the amount of freely accessible water on a substrate which is readily available for microbial growth. The a_w_ of pure water is 1.00 which equals to 100% relative humidity. Peanuts might be contaminated by aflatoxins if they are not dried immediately and fail to maintain a safe moisture level during post-harvest. According to Dorner (2008), inadequate drying of peanuts favors the growth of aflatoxigenic *Aspergillus* spp. during storage. This is in fact a challenge since peanuts are naturally hygroscopic and tend to absorb moisture from the surrounding storage environment (Waliyar et al., 2015a). Therefore, the source of moisture during storage such as leaking roofs and condensation due to improper ventilation in the warehouse should be avoided in order to maintain low moisture levels during storage. It is recommended to store peanuts with moisture content <7% and <9% for shelled and unshelled peanuts, respectively to avoid fungal growth. These moisture content levels might guarantee safe storage for peanuts for approximately 1 year if the temperature and relative humidity are maintained at 25 – 27°C and 70%, respectively (Torres et al., 2014; Waliyar et al., 2015a). According to Villers (2014), fungi start to grow when the relative humidity exceeds 65% during storage. Temperature and a_w_ has a significant effect on the growth of *Aspergillus* section *Flavi*, aflatoxin biosynthesis gene expression and the subsequent aflatoxin production (Schmidt-Heydt et al., 2009; Abdel-Hadi et al., 2012; Bernáldez et al., 2017). However, the minimum a_w_ for growth varies depending on the temperature and nutrient availability in the substrate. The minimum a_w_ for *A. flavus* growth was reported to be at 0.91 a_w_ at 25 and 37°C in sorghums (Lahouar et al., 2016), while the minimum a_w_ in paddy was predicted between 0.83 and 0.85 (Mousa et al., 2011). A similar range of minimum a_w_ was observed in shelled peanuts (Liu et al., 2017). The authors also demonstrated a lower growth rate when a_w_ < 0.85 or temperature < 20°C, while better growth was observed at a higher a_w_ and around 28–40°C.

The growth of *A. flavus* might occur over a wider range of temperature and a_w_ level as compared to the aflatoxin production which occur in a narrower range of conditions (Abdel-Hadi et al., 2012; Liu et al., 2017). According to Abdel-Hadi et al. (2012), the optimum temperatures and a_w_ level for *A. flavus* was 30 – 35°C and 0.99 a_w_. The marginal conditions for the growth were reported at 15 and 40°C at 0.99 a_w_. On the other hand, the optimum conditions for AFB_1_ production were 30 – 35°C at 0.95 a_w_, and 25 – 30°C at 0.99 a_w_. Another study by Schmidt-Heydt et al. (2010) reported that the growth of *A. parasiticus* was optimum at 35°C. However, AFB_1_ and AFG_1_ production were optimum at >37°C and 20 – 30°C, respectively. They also discovered that temperature was the key parameter for AFB_1_ production, whereas a_w_ contributed more to AFG_1_. The optimum temperature of *A. flavus* growth on shelled peanut was 37°C while the production of AFB_1_ was maximum at 28°C and 0.96 a_w_. AFB_1_ was not detected at a_w_ < 0.90 when temperature fell below 20°C or a_w_ ≥ 0.96 when the temperature was higher than 40°C (Liu et al., 2017).

Drought stress in the field was reported to increase the aflatoxin contamination in peanuts due to over-maturity, reduction of moisture content in seeds and increased risk of insect and pod damage which facilitate the aflatoxigenic *Aspergillus* spp. infection in peanuts (Craufurd et al., 2006; Waliyar et al., 2015b; Sibakwe et al., 2017). A previous study by Sibakwe et al. (2017) reported that severe drought caused poor growth and pod development which increased the susceptibility to *A. flavus* infection. In addition, the growth of *A. flavus* was supported by the exudation of sucrose from roots and peanut pods under the drought stress. Therefore, high levels of *A. flavus* and aflatoxins were recorded during prolonged drought. Another study by Arunyanark et al. (2009) demonstrated that high soil temperature and low moisture in soil favored aflatoxin production in peanuts. High soil temperature enhanced moisture loss from peanut kernel and subsequently reduced the a_w_ level. Low a_w_ in peanut kernels results in the reduction of phytoalexins which are responsible for the defense mechanism against plant pathogens.

## Peanut Production and Consumption in Malaysia

Peanuts are not the main agricultural product in Malaysia, and the local production was just around 231 tons in 2016 as compared to the main producer countries such as China (16,685,915 tons), India (6,857,000 tons), Nigeria (3,028,571 tons) and the United States (2,578,500 tons). In Southeast Asia, Indonesia, and Vietnam are the main peanut producers, which recorded a total production of 504,912 tons and 427,190 tons in 2016, respectively (FAOSTAT, 2017). Peanut production in Malaysia has declined since 1985 and since then, the import of peanuts has gradually increased and peaked in 2011 (FAOSTAT, 2017). As local peanut production is low, Malaysia needs to import peanuts from other countries in order to meet the local demand.

In Malaysia, peanuts are widely used as the raw material for local dishes and other peanut-based products such as peanut sauces, cookies, roasted peanuts, peanut butter and peanut snacks (Leong et al., 2010; Norlia et al., 2018b). However, from a food safety perspective, peanuts are known as a common food allergen and a carrier for foodborne diseases such as aflatoxicosis and salmonellosis (Chang et al., 2013). The presence of aflatoxins is among one of the crucial aspects that regulate the quality of peanuts other than the physical and chemical properties. Based on Malaysian Food Consumption Statistics (IPH, 2014), the mean daily intake of peanuts among Malaysian were 1.86 g/day (non-frequent eaters) and 4.95 g/day (frequent eaters), respectively. Generally, the Malays recorded the highest intake for both peanuts and peanut butter. Long term intake of aflatoxin-contaminated foods leads to a chronic exposure and hence increases the risk of hepatocellular carcinoma (HCC), commonly known as liver cancer. Several researchers have estimated the dietary exposure of aflatoxins among the Malaysian population (Leong et al., 2011; Chin et al., 2012). For AFB_1_, Chin et al. (2012) reported the dietary exposure of 24.3–34.0 ng/kg bw/day. Among 236 food composites tested, peanuts were found to be the main contributor to aflatoxin contamination. Based on this finding, the liver cancer risk among the Malaysian population was estimated to be 0.61 – 0.85% cancers/100,000 population/year which contributed to 12.4 –17.3% of the liver cancer cases.

## Adverse Effects of Aflatoxins to Humans and Animals

Aflatoxin exposure in humans could be due to direct or indirect consumption of contaminated foods. Direct exposure is when the aflatoxin-contaminated food is directly consumed while the indirect exposure is caused by the ingestion of dairy product contaminated with AFM_1_, or consumption of meat product from animals fed with contaminated feed. AFM_1_ has also been detected in human breast milk which subsequently exposes the baby to aflatoxins (Dhanasekaran et al., 2011). Aflatoxicosis is a health complication due to the ingestion of aflatoxin-contaminated foods. However, the response depends on the age and health condition, nutritional diet, level and duration of exposure, and environmental factors (Wagacha and Muthomi, 2008). The rapid onset and obvious toxic response are signs of acute toxicity of aflatoxins. Other symptoms of aflatoxicosis might include diarrhea, jaundice, low-grade fever, anorexia, and a decrease in the amount of essential serum protein, which is synthesized by the liver. In severe cases, aflatoxicosis might cause death to humans. Chronic aflatoxicosis results in cancer, immune suppression, stunted growth and malnutrition among children (Lewis et al., 2005; Wild and Gong, 2010).

The liver is known to be the main target for aflatoxin toxicity and carcinogenicity. The lesion could be observed in the affected liver, and this increases the risk of HCC over time (Liu and Wu, 2010). The HCC has been well documented, and the incidence is most likely to occur in a person with chronic hepatitis B virus (HPV) infection. In addition, children chronically exposed to aflatoxin-contaminated breast milk and other dietary foods, especially peanut-based product might develop cirrhosis especially in the malnourished ones (Dhanasekaran et al., 2011).

The consumption of aflatoxin-contaminated feed in animals also results in similar symptoms, and the susceptibility depends on age, species and individual variation. Acute aflatoxicosis may cause depression, weight loss, liver damage and gastrointestinal bleeding in animals while in severe cases, death may occur within several days. Prolonged aflatoxin exposure may reduce the growth rate of young animals and affect the quality of milk and egg due to the contamination of AFM_1_. The hepatic pathology in affected animals includes an enlarged gall bladder, changes of fatty acid in the hepatocytes, bile duct proliferation and diluted bile. In addition, AFB_1_ has also been reported to reduce the nutrient adsorption and causes immunosuppression in animals (Lizárraga-Paulín et al., 2011; Sarma et al., 2017).

## The Occurrence of Aflatoxins in Raw Peanuts and Peanut-Based Products

The warm temperature (28 – 31°C) and high humidity (70 – 80%) in Malaysia favor the growth of *Aspergillus* spp. and cause the peanuts to be easily deteriorated due to fungal infection when stored under these conditions. The occurrence of aflatoxigenic *Aspergillus* section *Flavi* in a variety of nuts, cocoa beans, coffee, grapes, rice, dried fruits, corn, and small grains has been extensively reviewed by Taniwaki et al. (2018). However, the occurrence of these species does not always result in aflatoxin contamination as they might be present in foods without producing any toxins. In relation to aflatoxins, some authors pointed out that, on average, 50% of the isolated strains were able to produce aflatoxins in food (Geisen, 1998). Many strategies on the mitigation of aflatoxin in peanuts, including physical, chemical and biological methods, have been discussed and reported (Dorner, 2008; Wagacha and Muthomi, 2008; Torres et al., 2014; Waliyar et al., 2015a). However, none of the method could entirely eliminate aflatoxins in the food commodities.

Aflatoxin contamination occurs during pre-harvest, post-harvest and worsens during storage at the granary. A previous study in Mali indicated that aflatoxin level increased with increasing storage period at the granary (Waliyar et al., 2015b). According to the authors, aflatoxin contamination occurred due to pest damage and the inappropriate storage conditions that favored the growth of aflatoxigenic *Aspergillus* spp. Another study in Malawi also demonstrated a similar trend in aflatoxin contamination during post-harvest (Monyo et al., 2012). Samples were collected from different districts in Malawi, and the results revealed that 21 and 8% of samples in 2008 and 2009 respectively, were contaminated with aflatoxin level higher than 20 ppb. Aflatoxins in peanut-based products have also been reported especially from the African and Asian countries. Table 1 summarizes the occurrence of aflatoxins in raw peanuts and peanut-based products from different countries. Most of the peanut-producing countries such as Kenya, Haiti, and Indonesia reported very high concentrations of aflatoxins in peanut based-products (Ambarwati et al., 2011; Ndungu et al., 2013; Schwartzbord and Brown, 2015). In contrast, other peanut-importing countries such as Taiwan (Chen et al., 2013) and Korea (Ok et al., 2007) recorded a lower level of aflatoxin concentration in their peanut-based products. A study by Matumba et al. (2015) revealed that aflatoxin levels in peanut-based products on the local market in Malawi were significantly higher as compared to the raw peanuts intended for exports. This crucially indicated that the non-compliant samples for exports were not removed from the domestic supplies probably due to the limited public awareness among the consumers. A similar finding was reported by Schwartzbord and Brown (2015) who found that 94% of the peanut butter samples were heavily contaminated with aflatoxins, with the majority of samples exceeding 20 μg/kg. In contrast, only 14% of the raw peanut samples exceeded the regulatory limit. This might indicate that the contamination occurred more during storage pre-processing as compared to post-harvest. Ezekiel et al. (2012) also reported high aflatoxin contamination level in peanut cakes marketed in Nigeria, with 90% of the samples exceeding 20 μg/kg for total aflatoxins.

**TABLE 1:** The occurrence of aflatoxins in peanuts from different countries.

**Country**	**Type of peanuts**	**No. of**	**Aflatoxin level**		**^∗^Non-compliant**	**References**
		**samples**	**(μg/kg)**		**samples (%)**	
			**Mean**	**Range**		
^a^Kenya (Nairobi and Nyanza)	Raw peanut	3	18.3	0.0 – 52.4	20	Ndungu et al., 2013
	Roasted peanut	8	54.8	2.4–297.7	50	Ndungu et al., 2013
	Peanut butter	11	318.3	0.0–2377.1	73	Ndungu et al., 2013
	Unsorted peanut	11	111.2	0.0–364.7	74	Ndungu et al., 2013
	Sorted peanut	4	24.0	0.0–82.4	18	Ndungu et al., 2013
^a^Kenya (Eldoret and Kericho)	Raw peanut	78	146.8	37.8–340.2	n.a.	Nyirahakizimana et al., 2013
	Roasted coated	101	56.5	29.4–93.1	n.a.	Nyirahakizimana et al., 2013
	Roasted de-coated	49	19.9	0.0–42.3	n.a.	Nyirahakizimana et al., 2013
Nigeria^b^	Peanut cake	29	200.0	10–2820	90	Ezekiel et al., 2012
Brazil	Raw peanut	48	12.9	n.a.	8.3	Oliveira et al., 2009
	Raw peanut	58	45.3	n.a.	n.a.	Hoeltz et al., 2012
	Peanut product	43	49.8	n.a.	n.a.	Hoeltz et al., 2012
	Ground candy peanut	48	9.0	n.a.	8.3	Oliveira et al., 2009
	Salty roasted peanut	48	1.6	n.a.	–	Oliveira et al., 2009
	Salty dragee peanut	48	3.32	n.a.	2.1	Oliveira et al., 2009
Malawi	Raw peanut (local market)	69	122.3	0–501.0	n.a.	Matumba et al., 2015
	Raw peanut (for export)	27	2.6	0–9.3	–	Matumba et al., 2015
	Peanut butter	14	72.0	34.2–115.6	n.a.	Matumba et al., 2015
^b^Haiti	Raw peanut	21	n.a.	2.0–787	14	Schwartzbord and Brown, 2015
	Peanut butter	11	n.a.	2.0–2720	82	Schwartzbord and Brown, 2015
Korea	Raw peanut	27	4.07	0.1–18.0	n.a.	Ok et al., 2007
	Peanut butter	19	3.6	1.3–6.4	n.a.	Ok et al., 2007
Taiwan	Raw peanut	257	14.9	0.3–107.1	0.8	Chen et al., 2013
	Peanut butter	142	2.8	0.2–32.5	4.9	Chen et al., 2013
^c^Thailand	Raw peanut	20	47.1	n.d.–303.6	5	Kooprasertying et al., 2016
	Raw peanut	28	102	4 - 576	n.a.	Lipigorngoson et al., 2003
	Peanut product	713	n.a.	0.7–3238	n.a.	Songsermsakul, 2015
	Roasted peanut	20	13.5	0.7–41.6	5	Kooprasertying et al., 2016
	Ground peanut	20	68.2	0.9–362.5	9	Kooprasertying et al., 2016
^d^Indonesia	Peanut products	15	8.0	0.4–53.1	13.3	Aisyah et al., 2015
	Roasted peanut	33	43.2	0–316.8	42	Ambarwati et al., 2011
	Flour-coated peanut	33	34.28	0–160	30	Ambarwati et al., 2011
	*Pecel/gado-gado* sauce	33	17.1	0–197.8	21	Ambarwati et al., 2011
	*Siomay* sauce	18	4.41	0–39.9	11	Ambarwati et al., 2011
	Peanut sauce	12	23.17	0–198.6	17	Ambarwati et al., 2011
	Roasted peanut	12	n.a.	0–204	n.a.	Razzazi-Fazeli et al., 2004
	Coated peanut	16	n.a.	5–870	n.a.	Razzazi-Fazeli et al., 2004
	Peanut cake	10	n.a.	5–302	n.a.	Razzazi-Fazeli et al., 2004
	Peanut sauce	12	n.a.	7–613	n.a.	Razzazi-Fazeli et al., 2004
	Peanut butter	10	n.a.	7–228	n.a.	Razzazi-Fazeli et al., 2004
^e^Malaysia	Raw peanut	6	146.5	0–537.1	33	Farawahida et al., 2017
	Raw peanut	6	6.1	0.6–19.3	n.a.	Afsah-Hejri et al., 2013a
	Raw peanut	9	2.0	2.2–6.4	-	Khayoon et al., 2012
	Raw peanut	13	4.25	1.47–15.3	n.a.	Reddy et al., 2011
	Raw peanut	77	n.a.	0.1 – > 50	21	Ali, 2000
	Raw peanut	84	11.3	0–103.2	10.7	Arzandeh et al., 2010
	Raw peanut	14	n.a.	17.8–711	n.a.	Leong et al., 2010
	Raw peanut	20	n.a.	0–33.4	n.a.	Hong et al., 2010
	Raw peanut	145	n.a.	0.85–547.5	45	Sulaiman et al., 2007
	Raw peanut	210	n.a.	0.3–762.1	n.a.	Abidin et al., 2003
	Peanut sauce	6	22	0–59.5	33	Farawahida, 2018
	Roasted peanut (in shell)	10	n.a.	29.7–179	n.a.	Leong et al., 2010
	Roasted peanut (shelled)	20	n.a.	40.1–46.0	n.a.	Leong et al., 2010
	Peanut butter	12	n.a.	16.6–67.3	n.a.	Leong et al., 2010
	Coated nut product	20	n.a.	113.0–514.0	n.a.	Leong et al., 2010
	Peanut butter	23	n.a.	0.1–35	17	Ali, 2000
	Other peanut product	74	n.a.	0.1–>50	26	Ali, 2000

*^∗^Maximum regulatory limit for total aflatoxins set by respective countries. ^*a*^Kenya Bureau of Standard (KEBS): 10 μg/kg. ^*b*^USDA maximum limit of total aflatoxins: 20 μg/kg. ^*c*^Thai National Bureau of Agricultural Commodity and Food: 20 μg/kg. ^*d*^Indonesian Regulation: 15 μg/kg (raw peanut), 20 μg/kg (peanut product). ^*e*^Malaysian Regulation (1985): 15 μg/kg (raw peanut), 10 μg/kg (peanut product). n.a., data not available. n.d., not detected.*

In Malaysia however, aflatoxin contamination was mostly reported in raw peanuts as compared to peanut-based products. Abidin et al. (2003) revealed that 92% of raw peanut samples collected from five districts in Perak were contaminated in the range of 0.3 – 762.1 μg/kg. Furthermore, about 42% of raw peanut samples collected from Kuala Terengganu were also contaminated with aflatoxins in the range of 0.2 – 101.8 μg/kg (Hong et al., 2010). In Selangor, Arzandeh et al. (2010) reported that about 78.5% from a total of 84 raw peanut samples collected from the retail market were contaminated, and about 10.7% of the samples exceeded the maximum tolerable limit. The aflatoxin concentrations varied from 2.76 to 97.28 μg/kg. Another study by Farawahida (2018) reported that aflatoxin contamination ranged from 12.8 – 537.1 μg/kg and 5.1 – 59.5 μg/kg in raw peanuts and peanut sauce, respectively. About 38 and 22% of raw peanut samples collected from the retailers and manufacturers in Malaysia respectively, were found to exceed the Malaysian Regulation limit (Norlia et al., 2018b). In addition, the authors reported that aflatoxin contamination in raw peanut samples ranged from <LOD – 1021.4 μg/kg, while peanut-based product samples recorded a lower level of contamination (<LOD – 19.4 μg/kg). However, there was no significant difference in the *Aspergillus* spp. contamination for both types of peanuts, and there was only a moderate relationship (Pearson’s *r* = 0.425, *p* = 0.00) between AFB_1_ and *A. flavus/A. parasiticus* count. According to Martins et al. (2017), the *Aspergillus* spp. count and aflatoxin amount in peanuts does not always positively and strongly correlate especially in processed peanuts. The reduced a_w_ in the dried peanut-based products reduces the levels of viable aflatoxigenic fungi as they rarely grow below 0.8 a_w_. However, the aflatoxins still remain in the products. According to Farawahida et al. (2017), a combination of oil-less frying of chili powder and retort processing of peanut sauces significantly reduced the aflatoxin concentration but could not entirely eliminate them from the products.

Aflatoxins in peanut-based products were also reported in samples collected from the local markets in Malaysia. In Penang, a total of 196 nuts and nut products were tested for aflatoxins, and 16.3% of these were contaminated with aflatoxins ranging from 16.6 to 711 μg/kg (Leong et al., 2010). Coated nut products were found to be the highest contaminated sample in the range of 113.0 – 514.0 μg/kg. Apart from that, a previous study by Ali (2000) also reported high contamination of aflatoxins in peanut butter (0.1 – 35 μg/kg), and a local traditional product called “*kacang tumbuk*,” which was prepared from blended peanut, was found to be the most contaminated product. Similar findings were also reported by researchers from the neighboring country, Indonesia (Ambarwati et al., 2011).

## *Aspergillus* spp. and Aflatoxin Contamination Along the Peanut Supply Chain

A food supply chain describes the processes involved from food production to food consumption which often includes processors, packers, distributors, transporters, retailers, and consumers (Levinson, 2009). For agricultural commodities, an efficient supply chain management is vital since these commodities are naturally susceptible to fungal invasion pre- and post-harvest, and as a result, aflatoxin contamination. The overall peanut supply chain consists of several major stakeholders which include the producers, collectors, shellers, exporters, importers, manufacturers, retailers, and finally the consumers (Archer, 2016). There are several stages for fungal contamination at post-harvest stage such as sun-drying and threshing, shelling, sorting, blanching and roasting. However, the manufacturing process varies depending on the types of its final product. For example, the process might include grinding, pressing, blending, heating, cooling, and packing.

Martins et al. (2017) reported that various fungi, such as *Fusarium* spp., *Penicillium* spp. and *Aspergillus* spp., were isolated from peanuts along the production chain. Drying is the most important step to reduce the a_w_ in peanuts in order to prevent fungal growth. Interestingly, apart from fungi, aflatoxins were also found throughout the peanut production chain. This indicated that even though the level of fungal contamination could be reduced upon drying, aflatoxins remained in the peanuts. Another study by Guezlane-tebibel et al. (2013) on imported peanuts from China marketed in Algiers reported that the *Aspergillus* section *Flavi* was the highest with 79.3% of the isolates being highly toxigenic. Three strains of *Aspergillus* section *Flavi* (*A. flavus*, *A. minisclerotigenes* and *A. caelatus*) were identified through the polyphasic approach which included morphological, chemical and molecular techniques. These results indicated that these species were able to survive and contaminate the imported peanuts.

**FIGURE 1:**
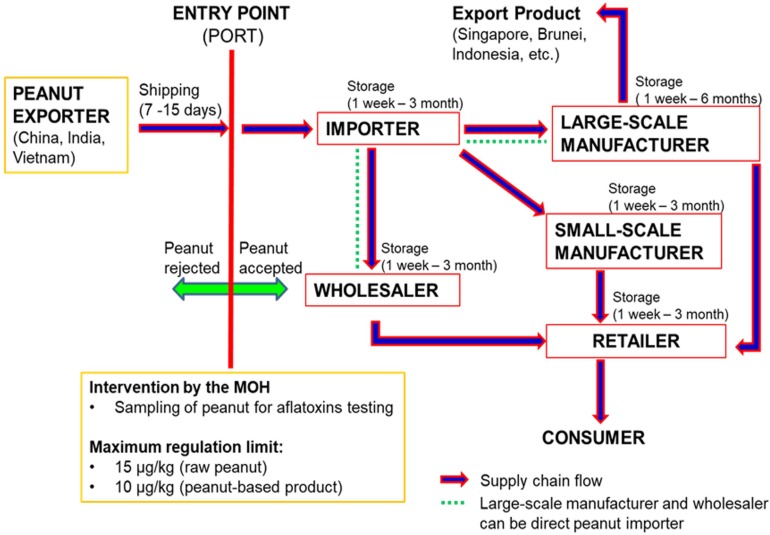
Peanut supply chain Malaysia (Source: Personal Communication with Malaysian Ministry of Health and Peanut’s stakeholders).

Figure 1 illustrates the flow of the peanut supply chain in Malaysia. The supply chain of imported peanut involves several major stakeholders, which are directly accountable and equally involved in handling the peanuts from entry at ports to the manufacturing industry, retailing and finally the consumers. The importers, manufacturers and retailers are the three main peanut stakeholders in the supply chain in Malaysia. To date, there is still lack of reports on the occurrence of aflatoxins in peanuts along the supply chain in Malaysia especially at the importer’s and manufacturer’s stages. The available data on the occurrence of aflatoxins in foodstuffs are mainly from the samples collected from the retailers, and most of the findings revealed high levels of aflatoxins especially in peanuts and peanut-based products (Ali, 2000; Abidin et al., 2003; Arzandeh et al., 2010; Leong et al., 2010; Reddy et al., 2011; Chin et al., 2012). Therefore, more investigations are required to identify the critical points of aflatoxin contamination along the peanut supply chain in Malaysia. Even though aflatoxin is not easily eliminated from the food supply chain, the information will be useful for use as a database in the development of intervention strategies to further reduce aflatoxins in foodstuffs.

Previous researches were only focusing on the peanut-producing countries especially in the African region (Mutegi et al., 2013; Wagacha et al., 2013). According to Waliyar et al. (2015a), the optimal bulk storage condition for peanut kernels at post-harvest stage was by maintaining the moisture content of <7.5%, relative humidity of 65% and temperature of 10°C. For the unshelled peanuts, higher moisture content (9%), relative humidity (70%), and temperature (25 – 27°C) could prevent the aflatoxigenic fungal growth and ensure a safe storage of peanuts for up to 1 year for export purposes. However, the optimal condition could not be maintained during shipping, transportation, and storage at the manufacturer’s or retailer’s premises due to the fluctuated temperature, inadequate ventilation and condensation which might occur along such processes (Wagacha and Muthomi, 2008). In this case, there is a possibility for re-emergence of the aflatoxigenic fungi in the peanuts once they reached the importing countries. Thus, it is important to identify and characterize the fungal species that could survive in the importing countries and evaluate their ability to re-produce the aflatoxins.

A recent study on *Aspergillus* spp. contamination and aflatoxins in imported raw peanuts and their products (produced locally using the imported raw peanuts) along the supply chain in Malaysia revealed that aflatoxins were absent in samples collected from the importer (Norlia et al., 2018b). However, the fungal contamination, especially from the *Aspergillus* section *Flavi* were high in these samples and not significantly different from other stakeholders (manufacturers and retailers). In contrast, aflatoxin contamination in raw peanuts was significantly higher in samples collected from the manufacturers and retailers. Their findings indicated that the aflatoxigenic *Aspergillus* spp. could survive in imported peanuts and start to grow and produce aflatoxins when the storage conditions at the manufacturer’ and retailer’s premises become favorable for their growth. The tropical climate with high temperature and humidity in this country easily deteriorates the stored peanuts and favors the growth of aflatoxigenic *Aspergillus* spp. Further identification and characterization of the isolates using the morphological, chemical and molecular approach confirmed the identity of the aflatoxigenic species as *A. flavus* (Norlia et al., 2018a, 2019).

## International Regulations of Aflatoxins and the Trade Impact on Peanut Supply Chain

Many countries have set the mycotoxin regulations to ensure the safety of the consumers and avoid the harmful effects of mycotoxins. These regulations are enforced by removing the non-compliant food products from the market (van Egmond et al., 2007). Based on the government regulations and guidelines in each country, both consumers and food processors could expect that aflatoxin level in foods should be below the disease-inflicting limits (Anukul et al., 2013). Aflatoxins were the first mycotoxin to be regulated (in the late 1960’s), and now the regulations have been set in approximately 100 countries around the world which cover approximately 85% of the world’s population (van Egmond and Jonker, 2004). The accessibility of the toxicological data and its incidence, socio-economic problems, and information on the sampling and analysis are the important aspects involved in the decision-making process of setting up the regulation limit (van Egmond and Jonker, 2004).

Internationally, the European Union (EU) regulation, US Food and Drug Administration (FDA) and Codex Alimentarius Commission (CAC) have been accepted as the guidelines for establishing the maximum regulatory limit for aflatoxins. Codex was co-founded by the Food and Agriculture Organization (FAO) and the World Health Organization (WHO) in 1963 with the objective to establish the Codex standards, guidelines, and Code of Practice for defending the health of consumers and verifying good practices in food trade. Generally, the aflatoxin regulatory limits are different in each country as shown in Table 2. Aflatoxins in peanuts are regulated in most of the countries since this commodity are naturally vulnerable to *Aspergillus* spp. infection and the subsequent aflatoxin contamination. European Union has the strictest regulations which allow only 2 μg/kg and 8 μg/kg of AFB_1_ in peanut products for direct human consumption and raw peanuts intended for further processing, respectively [Commission Regulation (EU) No. 165/2010], while Codex sets the maximum limit of total aflatoxins at 15 μg/kg (Codex Stan Cxs 193-1995, 1995). A maximum level of 20 μg/kg of total aflatoxins in peanuts has been enforced by the FDA^1^. Other countries mostly regulate the total aflatoxins in peanuts and peanut based-products with a maximum limit of 10 – 35 μg/kg except for Singapore (5 μg/kg). In this regard, Malaysia has set a maximum limit of 10 μg/kg and 15 μg/kg for total aflatoxins in ready-to-eat peanuts and raw peanuts intended for further processing, respectively (Food Act 1983, 2014). These regulations were established to help protect the consumers against the harmful effects of aflatoxin by preventing the compounds from entering the peanut supply chain in the country. Even though the current maximum regulatory limit was reported to be adequate in protecting Malaysians’ health against aflatoxin, the chronic exposure is still a concern (Chin et al., 2012).

**TABLE 2:** Aflatoxin regulatory limits in different countries.

**Country/**	**Type of**	**Type of**	**Maximum**
**Organization**	**aflatoxins**	**food**	**(μg/kg)**
European Union	AFB_1_	Peanuts	8
	Total aflatoxins	Peanuts	15
	AFB_1_	Peanut products	2
	Total aflatoxins	Peanut products	4
FDA	Total aflatoxins	Peanuts	20
Codex	Total aflatoxins	Peanuts	15
China	AFB_1_	Peanut, corn	20
Hong Kong	Total aflatoxins	Peanuts and peanut products	20
India	AFB_1_	All food	30
Indonesia	Total aflatoxins	All food	35
	AFB_1_	Peanut and corn	15
	Total aflatoxins	Peanut and corn	20
Japan	Total	All foods	10
South Korea	AFB_1_	Grains, cereal products	10
Malaysia	Total aflatoxins	Raw peanuts	15
	Total aflatoxins	Peanut products	10
Philippines	Total aflatoxins	All food	20
Singapore	Total aflatoxins	All foods	5
Sri Lanka	Total aflatoxins	All foods	30
Taiwan	Total aflatoxins	Peanut and corn	15
Thailand	Total aflatoxins	All foods	20
Vietnam	Total aflatoxins	All foods	10

*Source: Commission Regulation (EC) No. 165/2010 (2010) and Anukul et al. (2013), US Food and Drug Administration (FDA), Codex Stan Cxs 193-1995 (1995), Malaysian Regulation Food Act 1983 (2014).*

Nevertheless, the implementation of strict regulations may neither be a trade barrier nor a catalyst on the improvement of aflatoxin management (Emmott, 2012). This factor was the most important reason as to why sub-Saharan Africa and Malawi were stopped from exporting their peanuts to European countries, back in the late 1990s. These countries were losing their competitiveness and struggled to reach the stringent thresholds put in place. Only 40% of peanuts are directed to the core processing, wholesale and retail markets. Meanwhile, another 60% is locally consumed by farmers or sold directly by the producers on local markets (Emmott, 2012). According to Matumba et al. (2015), there are no other channels for diversion of the grade-outs to be exported and, hence, the peanuts are projected to only local market. Therefore, without proper aflatoxin management and control, this scenario will consequently affect the public which lacks the knowledge on aflatoxins. A survey conducted in Malawi discovered that information concerning aflatoxin was very restricted among the general public especially farmers (Matumba et al., 2015). Besides, the decline of the raw peanut export in most countries including Africa was also attributed to the internal supply side or macroeconomic, climatic shocks, market development, competitive cost, quality and sectoral-specific policies which subsequently reduced producer inducement through direct and indirect taxation (Rios and Jaffee, 2008).

## Aflatoxin Management in Peanuts Along the Supply Chain

Aflatoxins could not be easily eliminated from peanuts once they are formed. Hence, the aflatoxin management practices are important as the mitigation tools of aflatoxin contamination in the peanut supply chain. Proper prevention and management strategies of aflatoxins in peanuts during pre- and post-harvest stages has been suggested including lot segregation, density segregation, kernel moisture control, blanching, color sorting, and the use of biological control in the field (Dorner, 2008). Aflatoxin management strategies in the field have been described and reviewed extensively (Dorner, 2008; Torres et al., 2014; Waliyar et al., 2015a). Florkowski and Kolavalli (2014) reported on the application of soil amendments including the use of gypsum and compost as one of the strategies to reduce aflatoxins during pre-harvest. However, this method might not be economically feasible for farmers who are unable to commit and in return require higher yields to recover the additional production costs. Pandey et al. (2019) critically reviewed three pre-harvest mitigation alternative methods of aflatoxin by implementing genetic resistance for *in vitro* seed colonization (IVSC), pre-harvest aflatoxin contamination (PAC) and aflatoxin production (AP). The next-generation sequencing (NGS) technologies are believed to accelerate the advancement of genomic resources at a very reasonable cost even for large genome-polyploid crops including peanuts (Varshney et al., 2019).

Wood, bamboo, thatch or mud are commonly used by farmers as the storage structure for harvested peanuts. Poor storage practices is the main factor that leads to aflatoxigenic *Aspergillus* spp. infestation (Florkowski and Kolavalli, 2014). Although the aflatoxin regulation in each country could help to protect the consumers from the risk of aflatoxins in the imported peanuts, the presence of aflatoxigenic fungi might increase the risk of aflatoxin production and accumulation in peanuts during storage, especially at the manufacturer’ and retailer’s stages. However, the new storage practices including the use of metal or cement bins, polypropylene bags and hermetic packaging have been reported to improve the storage system and reduce aflatoxin contamination (Waliyar et al., 2015a). It is also important to retain low moisture level during storage, transportation and sales (Wagacha and Muthomi, 2008). Besides, the implementation of post-harvest machinery including threshers, dryers and shellers supports higher yield and lessens post-harvest processing and drying time. Physical separation or sorting also helps to remove the contaminated kernels by observing the physical appearances including color, size and density (Waliyar et al., 2015a).

It is the basic consumers’ right to consume safe and nutritious food products. Nevertheless, reports on the aflatoxin occurrence in peanuts on the Malaysian market found that some of the samples exceeded the maximum regulation limit (Arzandeh et al., 2010; Leong et al., 2010; Norlia et al., 2018b). Therefore, the cooperation between regulatory bodies, scientific communities and the industries is of utmost importance to promote and produce safe and quality foods (Anukul et al., 2013). The Malaysian government has enforced a strict regulation on aflatoxins in order to protect the consumers. Imported peanuts are screened for aflatoxins before they can be released to the local markets. The Malaysian Ministry of Health is responsible for conducting the screening of aflatoxins from the peanut consignment at the entry ports. The screening process involves peanut sampling and testing for aflatoxins. Any peanut consignment found to exceed the permissible limit will be rejected.

The involvement of private sectors in peanut-importing countries might also help in the management of aflatoxin issue along the supply chain. A previous study on the peanut stakeholders in Malaysia revealed that the hygiene and training program, knowledge on aflatoxins, storage practices and the quality assurance certification influence the hygiene practices required in minimizing aflatoxin contamination in peanut-based products (Azaman et al., 2016). It was also reported that the stakeholders who attended the training program on aflatoxin management applied better hygiene practices than those that did not attend any training programs. It was also found that the importers and large-scale manufacturers had a better knowledge and understanding of aflatoxin contamination as compared to the small-scale manufacturers and retailers. In Malaysia, most of the large-scale peanut manufacturers are certified with the Good Manufacturing Practice (GMP) and Hazard Analysis and Critical Control Point (HACCP) to ensure the safety of their products (Norlia et al., 2018b). A previous study by Farawahida (2018) revealed that aflatoxins in raw peanuts and peanut sauce samples obtained from the small and medium enterprises (SME’s) were more contaminated than the companies certified with GMP and HACCP.

Another study by Azaman et al. (2015) reported that the majority of food industry managers had a better knowledge of aflatoxins, and they recommended to provide relevant trainings to their food handlers and operators in order to further reduce aflatoxin contamination in peanut-based products. In this regard, peanut industries should only buy the raw materials from trusted suppliers which can provide the certification of aflatoxin analysis to ensure the safety of raw peanuts. The manufacturers can also have in-house validation of aflatoxin testing using the commercial aflatoxin testing kits to screen for aflatoxins in peanuts or other ingredients in peanut-based products such as spices. The involvement of the private sector in raising the public awareness on aflatoxin risk through public talks, trainings, fact sheets, social media and radio broadcasts might help to disseminate information and increase the knowledge among the peanut retailers and consumers as the majority of them are unaware of aflatoxin contamination (Sugri et al., 2017).

## Sampling, Detection and Quantification of Aflatoxins in Peanuts

A proper sampling procedure is crucial to obtain a representative sample that is valid for aflatoxin analysis. The variation in the amount of aflatoxins and the small percentage of contaminated kernels in a lot are the main challenges in sampling (Fonseca, 2002). The EU has published a guideline (Commission Regulation (EC) No. 401/2006, 2006) on the sampling and aflatoxin analysis for official controls of aflatoxins in imported peanuts and other types of nuts. The regulation is in line with the Codex sampling standard (Codex Stan Cxs 193-1995, 1995). In general, an aggregate sample of 20 kg is collected from 10 to 100 incremental samples collected at different sites and locations of the peanut lot. The samples are divided into two equal laboratory samples before grinding it for further analysis. The laboratory samples shall be mixed thoroughly to achieve complete homogenization. The lot will be rejected if the laboratory samples exceed the maximum limit of the permitted aflatoxins level after taking into account the correction for recovery and measurement of uncertainty. For sampling in storage structures (bins, sacks, containers), a suitable probe should be used to get a representative sample collected from different depths of the containers. Samples are taken at three different levels (bottom, middle and top) using a probe. Approximately 1 kg of total aggregate samples are randomly taken from each level, and mixed thoroughly before 1 kg of samples are taken for laboratory analysis (Mahuku et al., 2010).

The detection and quantification of aflatoxins in peanuts are usually based on their absorption and emission spectra. The AFB’s and AFG’s exhibit blue and green fluorescence at 425 and 540 nm under UV irradiation, respectively (Kumar et al., 2017). Thin Layer Chromatography (TLC) which is based on the visualization of fluorescent spots and their intensities is one of the oldest methods used for aflatoxin detection in peanuts (Younis and Malik, 2003; Bakhiet and Musa, 2011). Nowadays, more recent and advanced methods such as High Performance Liquid Chromatography (HPLC), Ultra-High Pressure Liquid Chromatography (UHPLC) and Liquid Chromatography Mass Spectroscopy (LC-MS) have been widely used in aflatoxin analysis (Afsah-Hejri et al., 2011; Ibáñez-vea et al., 2011; Sameni et al., 2014; Kumar et al., 2017). HPLC equipped with a fluorescence detector and C_18_ analytical column is the most frequent method cited in the literature for aflatoxin analysis in peanuts (Afsah-Hejri et al., 2011). This method, either with pre- or post-column derivatization, requires sample extraction with a mixture of methanol and water or chloroform and phosphoric acid, followed by the purification step using either the liquid-liquid extraction (LLE) (Bakhiet and Musa, 2011), solid phase extraction (SPE) (Khayoon et al., 2012) or immunoaffinity column (IAC). The IAC method is the most popular purification method for aflatoxins from peanuts used by researchers such as the AflaTest from Vicam (Afsah-Hejri et al., 2013b; Schwartzbord and Brown, 2015; Martins et al., 2017), and AflaPrep^®^ from R-Biopharm Rhone Ltd. (Magrine et al., 2011; Ruadrew et al., 2013).

Aflatoxin derivatization is required for aflatoxin analysis using a fluorescence detector to enhance the detection. Triflouro acetic acid (TFA) is used for pre-column derivatization (Khayoon et al., 2012) while post-column derivatization requires a Photochemical Reactor for Enhanced Detection (PHRED) which is attached adjacent to the HPLC analytical column (Afsah-Hejri et al., 2011). According to Soleimany et al. (2012), tandem mass spectrometry (MS/MS) has a high level of selectivity and could provide a higher degree of certainty in the identification of analytes. Besides, LC-MS or LCMS/MS techniques also enable the simultaneous detection and quantification of multi-mycotoxins at relatively low concentrations in various food products. Recently, UHPLC-MS/MS was used for multi-mycotoxin determination in peanuts (Sameni et al., 2014; Manizan et al., 2018).

Fast and easy-to-use methods for aflatoxin detection are required to facilitate the screening process. Rapid aflatoxin tests are being improved and allow the operators to carry out the test at point of purchase (*in situ*). In this regard, the immunochemical-based method such as Enzyme-Linked Immuno-Sorbent Assay (ELISA) is commonly used for aflatoxin screening in peanuts as the ELISA test kit for commercial application requires only a simple extraction method (Lipigorngoson et al., 2003; Mutegi et al., 2009; Leong et al., 2010; Aisyah et al., 2015). Many researches on the development and optimization of the monoclonal antibody’s performance in terms of sensitivity and cross-reactivity have been done to improve the method (Oplatowska-Stachowiak et al., 2016). A precise test kit based on the concept of lateral flow immunoassay can be used during field inspection and gives results within 5–15 min (Chen et al., 2016; Yu et al., 2018). It is very important to acquire high assay sensitivity as well as optimum immune-parameters. These testing kits have the potential to be a commercially viable intervention.

Immunosensor, a type of biosensor, is another alternative method for aflatoxin detection. Biosensor is an analytical instrument which combines the use of biological components (e.g., antibodies, nucleic acids, enzymes, cells, etc.) with a physicochemical transducer (Mosiello and Lamberti, 2011). Based on the same approach of the established analytical methods such as ELISA, many researchers aimed to transfer the method of the immunological assay from microtiter plates into a biosensor format (Azri et al., 2018). The developed electrochemical immunosensor showed a dynamic working range within 0.0001–10 μg/L, and the detection in spiked peanut samples provided a good recovery of between 80 and 127% (Azri et al., 2018).

The screening of aflatoxins might be a barrier to the peanut stakeholders primarily because of the testing cost and the need of a trained analyst to carry out the test. However, there are many other potential savings associated with aflatoxin screening at the point of purchase such as by ceasing the purchase of contaminated peanuts and lowering the processing cost by separating the highly contaminated peanuts from the good ones (Emmott, 2012).

## Molecular Identification and Characterization of *Aspergillus* section *Flav*i

The traditional method of isolation and cultivation using selective media are frequently used for the detection and identification of aflatoxigenic fungi. However, these methods are laborious, time-consuming and require taxonomical expertise as it is difficult to correctly identify based on morphological characteristics alone, especially those that are closely related (Rodrigues et al., 2009; Reis et al., 2014). Afsah-Hejri et al. (2013b) reported on the occurrence of aflatoxigenic *A. flavus* in peanuts from Malaysia but only based on the morphological identification. Besides, a similar study was reported by Reddy et al. (2011) on the occurrence of *Aspergillus* spp. in various food products marketed in Malaysia based on morphological identification. Morphology alone is insufficient and unreliable to correctly identify and differentiate the closely-related species within *Aspergillus* section *Flavi*. Therefore, the chemical profile of *Aspergillus* spp. is often used to assist the morphological identification (Rodrigues et al., 2009; Baquião et al., 2013). According to Samson et al. (2006), aflatoxins, aspergillic acid and cyclopiazonic acid are the main extrolites that are commonly used for the identification of aflatoxigenic *Aspergillus* spp. from section *Flavi.* Table 3 shows the common morphology, extrolites, and molecular identification which have been used as the major parameters to differentiate these species.

**TABLE 3:** Morphology, extrolite production and molecular identification of *Aspergillus* section *Flavi* species.

**Species**	**Morphology**	**Extrolites**	**Molecular**	**Origin**	**References**
			**identification**		
*A. flavus*	Yellow-green conidia, small and large sclerotia, orange reverse on AFPA	AFB (+/−), CPA (+/−), aspergillic acid, asperfuran (+/−), paspalinin and paspaline (+/−)	β-tubulin and calmodulin	*Arachis* hypogaea	Pildain et al., 2008
*A. parasiticus*	Dark-green conidia, orange reverse on AFPA	AFB, AFG, kojic acid, aspergillic acid, parasiticolides, paspalinin and paspaline (+/−)	β-tubulin and calmodulin	*Arachis hypogaea, A. vilosa, A. correntina*	Pildain et al., 2008
*A. nomius*	Yellow green conidia, orange reverse on AFPA	AFB, AFG, kojic acid, aspergillic acid, nominine	β-tubulin and calmodulin	Wheat	Pildain et al., 2008
*A. pseudonomius*	n.a	AFB, kojic acid, chrysogine	ITS, β-tubulin and calmodulin	Diseased alkali bees	Varga et al., 2011
*A. bombycis*	Yellow-green conidia, orange reverse on AFPA	AFB, AFG, kojic acid, aspergillic acid	β-tubulin and calmodulin	Frass in a silkworm rearing house	Pildain et al., 2008
*A. tamarii*	Dark-brown conidia, dark brown reverse on AFPA	Kojic acid, CPA (+/−),	β-tubulin and calmodulin	*Arachis hypogaea*	Pildain et al., 2008
*A. pseudotamarii*	Dark-brown conidia, dark brown reverse on AFPA	Kojic acid, AFB, CPA (+/−)	β-tubulin and calmodulin	Soil	Pildain et al., 2008
*A. caelatus*	Dark-brown conidia, dark brown reverse on AFPA	Kojic acid, CPA (+/−),	β-tubulin and calmodulin	Soil	Pildain et al., 2008
*A. pseudocaelatus*	n.a	AFB, AFG, kojic acid, CPA	ITS, β-tubulin and calmodulin	*Arachis bukartii*	Varga et al., 2011
*A. minisclerotigenes*	Yellow-green conidia, small sclerotia, orange reverse on AFPA	AFB, AFG, CPA, kojic acid, aspergillic acid, parasiticolides, aflavarins, paspalinin and paspaline, aflatrems and aflavinines	β-tubulin and calmodulin	*Arachis hypogaea*, soil, and peanut field	Pildain et al., 2008
*A. arachidicola*	Dark-green conidia, orange reverse on AFPA	AFB, AFG, aspergillic acid, kojic acid, parasiticolides, chrysogine	β-tubulin and calmodulin	*Arachis glabrata*	Pildain et al., 2008
*A. toxicarius*	n.a	n.a	β-tubulin and calmodulin	*Arachis hypogaea*	Pildain et al., 2008
*A. parvisclerotigenus*	Yellow-green conidia, orange reverse on AFPA	Kojic acid, AFB, AFG, CPA, aspergillic acid, aflavarins, paspalinin and paspaline, aflatrems and aflavinines	β-tubulin and calmodulin	*Arachis hypogaea*	Pildain et al., 2008
*A. korhogoencis*	Yellow-green to brown conidia, small sclerotia, orange reverse on AFPA	AFB, AFG, kojic acid, CPA, aspergillic acid, aflatrem, leporins, asparasone, aflavarin, aflavinine, paspalinin, and paspaline	ITS, benA, cmdA, mcm7, amdS, rpb1, preB, ppgA, and preA	*Arachis hypogaea*	Carvajal-campos et al., 2017
*A. leporis*	Yellow-green conidia	Kojic acid	β-tubulin and calmodulin	dung of *Lepus townsendii*	Pildain et al., 2008
*A. oryzae*	Yellow-green conidia	Kojic acid, asperfuran, aspirochlorin	β-tubulin and calmodulin	Unknown source, Japan	Varga et al., 2011
*A. sojae*	Yellow-green conidia	Kojic acid, aspergillic acid, asperfuran, aspirochlorine	β-tubulin and calmodulin	Soy sauce	Varga et al., 2011
*A. avenaceus*	n.a	Aspirochlorine	ITS, β-tubulin and calmodulin		Varga et al., 2011

*n.a, data not available. AFPA, *Aspergillus flavus* and *parasiticus* Agar. AFB, Aflatoxin B. AFG, Aflatoxin G. CPA, Cyclopiazonic acid. ITS, Internal Transcribed Spacer. +, present; −, absent.*

Nowadays, the molecular approach is widely used to accurately identify and describe the species in the genus *Aspergillus* especially when introducing a new species (Peterson, 2008; Frisvad et al., 2019). DNA sequence analysis of certain regions, such as the internal transcribed spacer (ITS), β-tubulin, calmodulin, and the aflatoxin gene cluster, has been analyzed to get information regarding the phylogenetic relationship among the species in this section (Pildain et al., 2008; Varga et al., 2011). However, none of them used a single approach to solve the identification problem. A polyphasic approach, which includes the morphological, chemical and molecular characteristics, is often used to identify and characterize the *Aspergillus* spp. in this section (Baquião et al., 2013; Reis et al., 2014). Godet and Munaut (2010) successfully identified nine species within the *Aspergillus* section *Flavi* using a six-step of molecular strategy including real-time PCR, RAPD and *SmaI* digestion. The results were validated by the partial sequencing of the calmodulin gene to confirm the identification.

The nuclear ribosomal DNA (rDNA) of the ITS region is the most widely sequenced region and recommended as the DNA barcoding marker for fungal identification at and below the genus level as well as the source of phylogenetic information. It is therefore necessary to include the ITS sequences whenever a new fungal species is described (Schoch et al., 2012). The ITS region is situated between the 18S (SSU) and 28S (LSU) genes in the rDNA repeat unit which includes the ITS1 and ITS 2 regions, and separated by the 5.8S gene. Of its three sub-regions, ITS1 and ITS2 are typically species specific and show a high rate of evolution (Nilsson et al., 2009). The entire sequence of the ITS region typically ranged from 450 to 700 bp. The amplification of the entire or part of the ITS region has been done by using various primers with the most commonly used primers were published by White et al. (1990).

Nevertheless, secondary identification markers, such as β-tubulin and calmodulin genes, are still needed to accurately identify *Aspergillus* section *Flavi* as ITS alone is still insufficient for molecular identification purposes (Samson et al., 2014). β-tubulin is a protein-coding gene that encodes for the tubulin protein which can be found in all eukaryotic cells as an elementary sub-unit of the microtubules. It involves in the eukaryotic cellular processes, and represents the main components of the cytoskeleton and eukaryotic flagella (Einax and Voigt, 2003). Calmodulin (CaM) is a calcium-binding protein that involves in the cell proliferation and differentiation in eukaryotic cells. It is highly conserved and serves as the main receptor for intracellular calcium (Ma et al., 2009). These three genes are widely used as the DNA markers for the identification and phylogenetic analysis of *Aspergillus* spp.

*A. arachidicola* and *A. minisclerotigenes* are the examples of two new aflatoxin-producing species in *Aspergillus* section *Flavi* that have been isolated from different species of peanuts and identified using phenotypic and molecular (β-tubulin and calmodulin gene sequences) characters (Pildain et al., 2008). Another new species in this section, *A. pseudotamarii*, has been described by Ito et al. (2001) by comparing the morphology, mycotoxin production, and divergence in ITS, 28S, β-tubulin and calmodulin gene sequences with the closely related species *A. tamarii* and *A. caelatus*. Besides, Tam et al. (2014) reported that the ITS, β-tubulin and calmodulin gene sequencing had successfully resolved the misidentification of *A. nomius* and *A. tamarii* from clinical isolates which were previously identified as *A. flavus* based on the morphological characteristic. However, this method could not be used to differentiate between the aflatoxigenic and non-aflatoxigenic species of *A. flavus* (Norlia et al., 2019). The aflatoxin biosynthesis gene cluster are present exclusively in the aflatoxigenic *Aspergillus* spp. such as *A. flavus* and *A. parasiticus*. The full cluster of aflatoxin biosynthesis genes has been characterized by Yu et al. (2004) and specific primers can be used to amplify the genes by using the PCR-based detection method (Erami et al., 2007). However, the identification of aflatoxigenic species could not be confirmed by this method as other genes that have not been tested might have defects or mutations that are not detectable by the specific primers. Takahashi et al. (2002) reported that deletion and other genetic flaws might have disrupted the aflatoxin pathway in both species. According to Abdel-Hadi et al. (2012), the gene expression and the aflatoxin production were affected by the temperature and a_w_. Therefore, the aflatoxin biosynthesis pathway can either be fully inhibited or activated depending on the environmental factors.

## Conclusion

Contamination of *Aspergillus* section *Flavi* and aflatoxins could occur at any stage along the peanut supply chain, specifically from the pre- and post-harvest stage at the producing countries to the peanut manufacturers and retailers at the importing countries. The high temperature and humidity in the tropical regions causes the inability to maintain the low moisture/a_w_ level of peanuts during storage, which subsequently enhances the growth of aflatoxigenic *Aspergillus* spp. especially *A. flavus*. Due to these reasons, the imported peanuts that are initially free from aflatoxins could be re-contaminated during the storage period at the manufacturers’ and retailers’ premises. Regular screening on the aflatoxins and aflatoxigenic *Aspergillus* spp. in peanuts should be regularly conducted to ensure that the stored peanuts are safe from the risk of aflatoxins. Various methods for aflatoxin and *Aspergillus* spp. screening, detection and quantification have been reviewed herein. The aflatoxin regulation in each country might help in protecting the population from the risk of aflatoxins but it does not guarantee the post-contamination after it enters the importing countries. Thus, aflatoxin management in peanut supply chain is very important and should involve both the government and private sectors. In addition, the awareness and knowledge on aflatoxins should be instilled among the peanut stakeholders and consumers to ensure that good handling and hygiene practices are applied during the storage of peanuts. Besides, the storage facilities, structures and conditions at the importing countries should also be taken into consideration in reducing the risk of aflatoxin contamination.

## Author Contributions

MN, MN-K, NS, and FA participated in the preparation of the manuscript. SJ and SR critically revised the manuscript and participated in the final editing of the manuscript.

## Conflict of Interest

The authors declare that the research was conducted in the absence of any commercial or financial relationships that could be construed as a potential conflict of interest.

## Footnotes

1 https://www.fda.gov/media/72073/download
